# Pseudomonas Synergizes with Fluconazole against *Candida* during Treatment of Polymicrobial Infection

**DOI:** 10.1128/iai.00626-21

**Published:** 2022-03-15

**Authors:** Siham Hattab, Anna-Maria Dagher, Robert T. Wheeler

**Affiliations:** a Department of Molecular & Biomedical Sciences, University of Maine, Orono, Maine, USA; b Graduate School of Biomedical Sciences and Engineering, University of Maine, Orono, Maine, USA; Tulane School of Medicine

**Keywords:** *Candida*, *Pseudomonas aeruginosa*, fluconazole, mucosal, polymicrobial, zebrafish

## Abstract

Polymicrobial infections are challenging to treat because we don’t fully understand how pathogens interact during infection and how these interactions affect drug efficacy. Candida albicans and Pseudomonas aeruginosa are opportunistic pathogens that can be found in similar sites of infection such as in burn wounds and most importantly in the lungs of CF and mechanically ventilated patients. C. albicans is particularly difficult to treat because of the paucity of antifungal agents, some of which lack fungicidal activity. In this study, we investigated the efficacy of anti-fungal treatment during C. albicans-P. aeruginosa coculture *in vitro* and co-infection in the mucosal zebrafish infection model analogous to the lung. We find that P. aeruginosa enhances the activity of fluconazole (FLC), an anti-fungal drug that is fungistatic *in vitro*, to promote both clearance of C. albicans during co-infection *in vivo* and fungal killing *in vitro*. This synergy between FLC treatment and bacterial antagonism is partly due to iron piracy, as it is reduced upon iron supplementation and knockout of bacterial siderophores. Our work demonstrates that FLC has enhanced activity in clinically relevant contexts and highlights the need to understand antimicrobial effectiveness in the complex environment of the host with its associated microbial communities.

## INTRODUCTION

Opportunistic microbes co-inhabit diverse host niches, leading to difficult-to-treat co-infections of immunocompromised individuals. However, we know little about how host tissue and microbe-microbe interactions affect antimicrobial sensitivity. Candida albicans and Pseudomonas aeruginosa are two of the most prolific opportunistic pathogens in the developed world, inhabit the same sites and are associated with polymicrobial infections (1–3). *Candida* is the fourth most common nosocomial pathogen and Pseudomonas is also associated with significant mono-microbial disease (4–6). *Candida*-Pseudomonas co-infections are associated with exacerbated disease, but it is not clear if co-infection should be treated with the same antimicrobials as mono-infection (7–10).

C. albicans and P. aeruginosa co-colonize numerous sites on the human body, including the gut, lungs, burn wounds, genitourinary tract, but most importantly they can be co-isolated in the lungs of cystic fibrosis (CF) patients (11, 12). CF is a genetic disease characterized by poor mucus clearance in the respiratory tract that leads to persistent infections and polymicrobial biofilms. P. aeruginosa infects around 70% of CF patients by the age of 30, and C. albicans is isolated in 75% of CF patients (13). Simultaneous colonization by these two pathogens has been linked to more severe clinical outcomes, due to accelerated decline in lung function and worsening of disease progression (7–10). However, the mechanism(s) underlying the postulated enhanced virulence are unknown, so it is difficult to determine if and how this interkingdom dialog regulates pathogenesis and therapy.

Co-infection of *Candida* with diverse bacteria leads to enhanced virulence (11, 14). C. albicans and P. aeruginosa interact through physical association, secreted factors and signaling molecules that can modulate important virulence factors in both pathogens. *In vitro* studies suggest that antagonistic interactions take place between P. aeruginosa and C. albicans through phenazines, ethanol and quorum sensing molecules (15–19). Diverse *in vivo* studies of *Candida*-Pseudomonas co-infection have shown either enhanced or decreased virulence (12, 15, 17–20). These *in vivo* studies suggest that a sophisticated understanding of the consequences of co-infection should account for multiple factors such as host environment, nutrient availability and host immune response that might shape these interactions.

C. albicans and P. aeruginosa have diverse strategies to sense host-relevant cues and adapt their cellular responses based on nutrient availability in the host (21–24). Micronutrient acquisition is a crucial aspect of virulence for most pathogens, including *Candida* and Pseudomonas (21, 25). Niche-specific levels of iron even lead to differential dependence on iron sensing and response machinery for *Candida*, with iron-rich environments requiring detoxification and iron-poor environments requiring enhanced acquisition (21, 26). Interestingly, iron starvation has been linked to increased antimicrobial susceptibility *in vitro* (27, 28). Understanding different pathways controlling these adaptation strategies will reveal new opportunities for novel therapeutic targets and more effective uses of existing antimicrobials.

Previous studies have predominantly focused on physical and molecular interactions between C. albicans and P. aeruginosa and their effect on growth, morphology and virulence, but little is known about effects of cohabitation with antimicrobial treatment. While mixed biofilms enhance antibacterial effects, it is relatively unexplored how these fungal-bacterial interactions affect antifungal drug efficacy during infection (29–31). Fluconazole (FLC) is highly effective and widely used in clinical settings to treat and prevent fungal infections, but paradoxically acts as a fungistatic drug *in vitro* (32). FLC tolerance is high among some clinical isolates and is associated with empirical treatment failure and worse outcomes (33), suggesting that reducing tolerance with adjuvant therapy may boost treatment success. Tolerance is frequently measured as trailing growth and manifests as slow *in vitro* growth of C. albicans in the presence of FLC at concentrations above the MIC, or MIC_50_. Fungicidal activity can be achieved *in vitro* with the addition of drugs such as HMG-CoA reductase inhibitors, calcineurin inhibitors, phenazines or iron chelators (27, 28, 33–39). Since microbes naturally produce these types of inhibitors, this raises the possibility that co-colonization or co-infection can produce conditions that enhance FLC activity.

To investigate if C. albicans-P. aeruginosa interactions affect FLC efficacy, we studied its activity *in vitro* and in the zebrafish infection model. Zebrafish is a powerful model organism that offers the advantage of examining infection outcomes *in vivo* while monitoring host and pathogen physiology through high resolution imaging (40, 41). The swimbladder is similar to the human lung, in that they are both air-filled, have a single layer epithelial lining that produces surfactant, and they share similar gene expression patterns (42–46). These similarities make the swimbladder infection model a useful tool to study mucosal infections (15, 47–51).

Previously, we found that P. aeruginosa and C. albicans are synergistically virulent in the swimbladder model, with enhanced invasive C. albicans growth and increased fish mortality (15). In this work, we investigated if FLC efficacy is modulated by P. aeruginosa during coculture and co-infection. Surprisingly, we observed that the combination of P. aeruginosa and FLC is synergistic against C. albicans, making the drug fungicidal and increasing its efficacy by over 3-logs. This striking effect was seen both *in vitro* and *in vivo.* Interestingly, iron supplementation led to a partial reversal of this synergy *in vitro* and *in vivo*. Taken together, these results suggest that the presence of co-colonizing or co-infecting microbes can substantially affect drug susceptibility in the vertebrate host.

## RESULTS

### Fluconazole is synergistic with P. aeruginosa against C. albicans *in vitro*.

**FIG 1:**
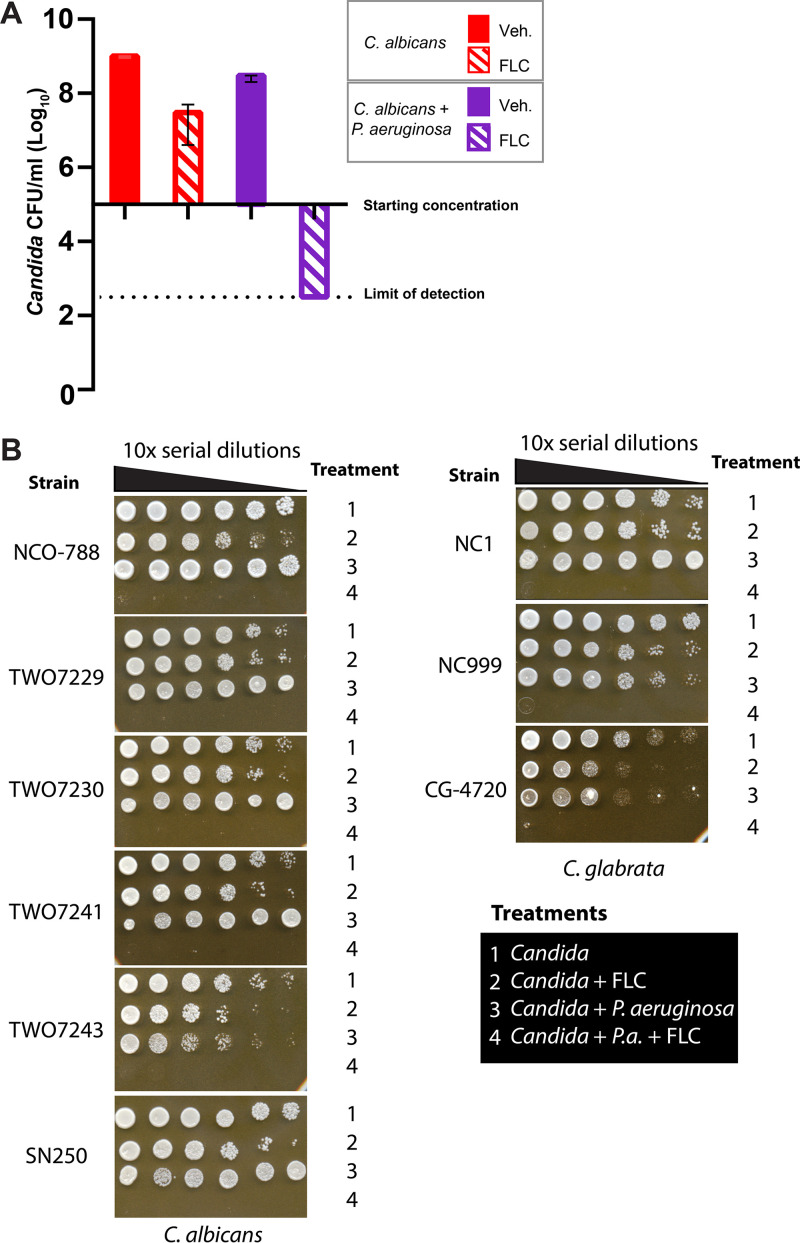
Fluconazole is synergistic with P. aeruginosa against C. albicans and C. glabrata *in vitro*. (A) C. albicans *+* P. aeruginosa + FLC shows a fungicidal effect after coculture. P. aeruginosa and C. albicans were inoculated at 2 × 10^5^/mL and FLC was added at 12.5 μg/mL. Drops (3 μL) of serial 10x dilutions of cocultures were plated on YPD containing antibiotics. Representative of >20 independent experiments. (B) Fungicidal effect of P. aeruginosa with FLC for multiple FLC-resistant and -sensitive clinical isolates of C. albicans and C. glabrata when tested at >2x MIC_50_. MIC_50_ was tested separately and supra-MICs of FLC were used (per measurements relevant for our assays; Table S1). Representative results of at least three independent experiments are shown.

C. albicans and P. aeruginosa are common opportunistic pathogens that are found in co-infections at multiple body sites, especially in the lungs of cystic fibrosis patients. We understand little about how co-infection affects virulence or whether treatment should be customized when both bacterium and fungus are co-isolated (13, 31). To determine if interactions between these microbes affect antimicrobial sensitivity, we performed coculture experiments to test if P. aeruginosa affects the antifungal action of fluconazole (FLC) against C. albicans. While FLC is fungistatic *in vitro* against C. albicans, in C. albicans - P. aeruginosa coculture it had potent fungicidal activity. FLC alone slowed growth of C. albicans while P. aeruginosa alone showed little to no effect on C. albicans growth, however the combination led to killing of greater than 1000x from the initial fungal inoculum (Fig. 1A). This loss of fungal viability is a hallmark for loss of FLC tolerance. Several FLC hyper-resistant C. albicans clinical isolates are also susceptible to this FLC*-*P. aeruginosa combination when supra-MICs of FLC are used (Fig. 1B). Synergy was also seen for susceptible and resistant clinical isolates of C. glabrata, which is evolutionarily distinct from C. albicans and has intrinsic FLC resistance (Fig. 1B). Fungicidal synergy was not observed with heat-killed bacteria (Fig. S1). The enhancement of FLC activity was reproducible in other media (YPD + serum) and at different temperatures (30°C, 37°C) (Fig. S2). Although these results argue for a robust bacteria-drug synergy, no *in vitro* conditions can truly substitute for the dynamic immune and nutritional environment found during infection.

### P. aeruginosa enhances fluconazole activity against C. albicans during swimbladder infection in zebrafish.

**FIG 2:**
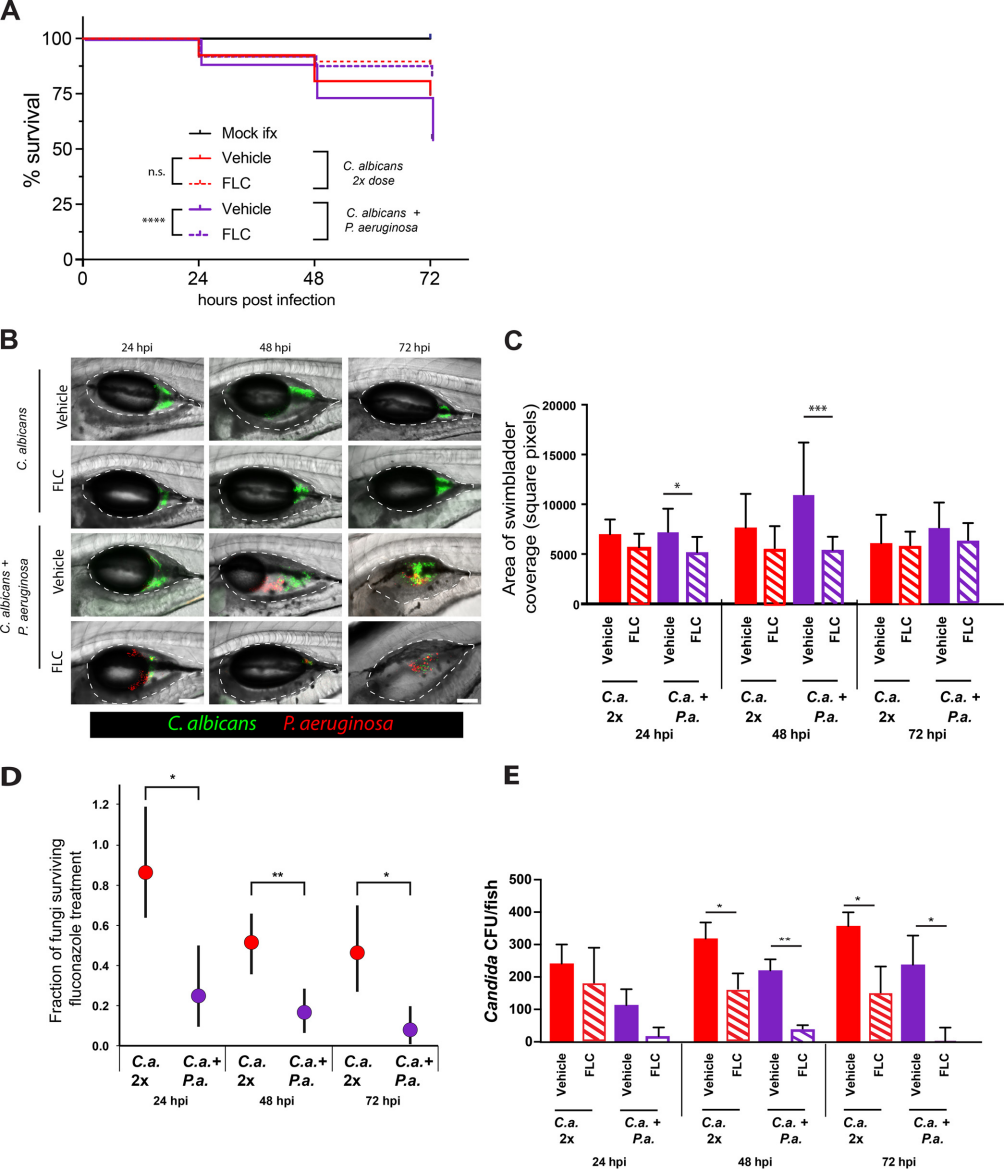
Fluconazole is synergistic with P. aeruginosa against C. albicans during mucosal infection. (A–E) Fish were infected in the swimbladder with either 50–100 C. albicans (mono-infection) or with 25–50 C. albicans and 25–50 P. aeruginosa, screened for fungal inoculum, then reared in water with or without 100 μg/mL FLC. (A) FLC-treatment increases survival during co-infection. Data pooled from 13 independent experiments. (B) Representative images of swimbladder infected with C. albicans or C. albicans + P. aeruginosa with or without FLC (100 μg/mL). Scale bars = 100 μm. Dotted white lines mark the boundary of the swimbladder. (C) C. albicans burden was measured by analysis of confocal z-stacks and calculation of square pixel coverage area. Graphs show medians and 95% confidence intervals. Data from 13 independent experiments. (D) Fraction of fungi surviving FLC treatment. Results are from 5 independent experiments. Monte-Carlo analysis was used to compare groups. (E) C. albicans burden calculated by CFU. Data from 5 independent experiments. (*p* > 0.05 NS; < 0.05 *; <0.01 **; <0.001 ***; <0.0001 ****).

To further examine C. albicans*-*P. aeruginosa interactions in the presence of FLC *in vivo*, we leveraged our zebrafish swimbladder co-infection model. This mucosal co-infection mimics conditions similar to human lungs and leads to synergistic virulence through enhanced fungal invasiveness (15, 50). To induce similar levels of mortality in mono- and co-infection, larvae were either mono-infected with a double dose of C. albicans (50–100 cells/fish) or co-infected with C. albicans (25–50 cells/fish) plus P. aeruginosa (50 cells/fish). We found that FLC treatment significantly reduced mortality in co-infection, although there was only a trend toward reduced mortality in the mono-infected group (Fig. 2A). This difference is reflected in different hazard ratios for FLC treatment in monoinfection (0.446, 95% CI 0.257–0.775) and co-infection (0.3255, 95% CI 0.243–0.437). This indicates that FLC is more effective in treating C. albicans*-*P. aeruginosa co-infection than fungal mono-infection, suggesting that there is also bacterial-drug synergy *in vivo*.

The enhanced survival of FLC-treated co-infected fish could be due to effects on the fungus, the bacteria and/or the host. We found that FLC does not affect zebrafish health (Fig. S3) or P. aeruginosa growth *in vitro* (Fig. S4). To test if decreased mortality is due to a decrease in C. albicans burden, fish were imaged by confocal microscopy at 24, 48, and 72 hpi, and we found fewer fluorescent C. albicans cells when co-infections were treated with FLC (Fig. 2B). This burden was quantified by counting fluorescent C. albicans pixels in the swimbladder. By this measure, FLC caused no significant decrease in C. albicans burden in mono-infected fish, but it caused a significant reduction in co-infected fish at 24 hpi and 48 hpi (Fig. 2C). Additionally, we homogenized fish and measured the number of viable C. albicans CFU per fish. The fraction of fungi surviving FLC treatment was strikingly higher during mono-infection compared to the co-infection, while there were almost no viable fungi in the co-infected fish treated with FLC (Fig. 2D and 2E). This CFU data is particularly robust, as FLC inhibits hyphal formation (52–54) and the process of homogenization biases against fungal hyphae, due to their strong inter-hyphal adherence and connections which tend to err on the side of undercounting. Together, these data suggest that the combination of FLC and P. aeruginosa have a fungicidal effect against C. albicans both *in vitro* and *in vivo*.

### Fluconazole - P. aeruginosa synergy is associated with iron limitation.

**FIG 3:**
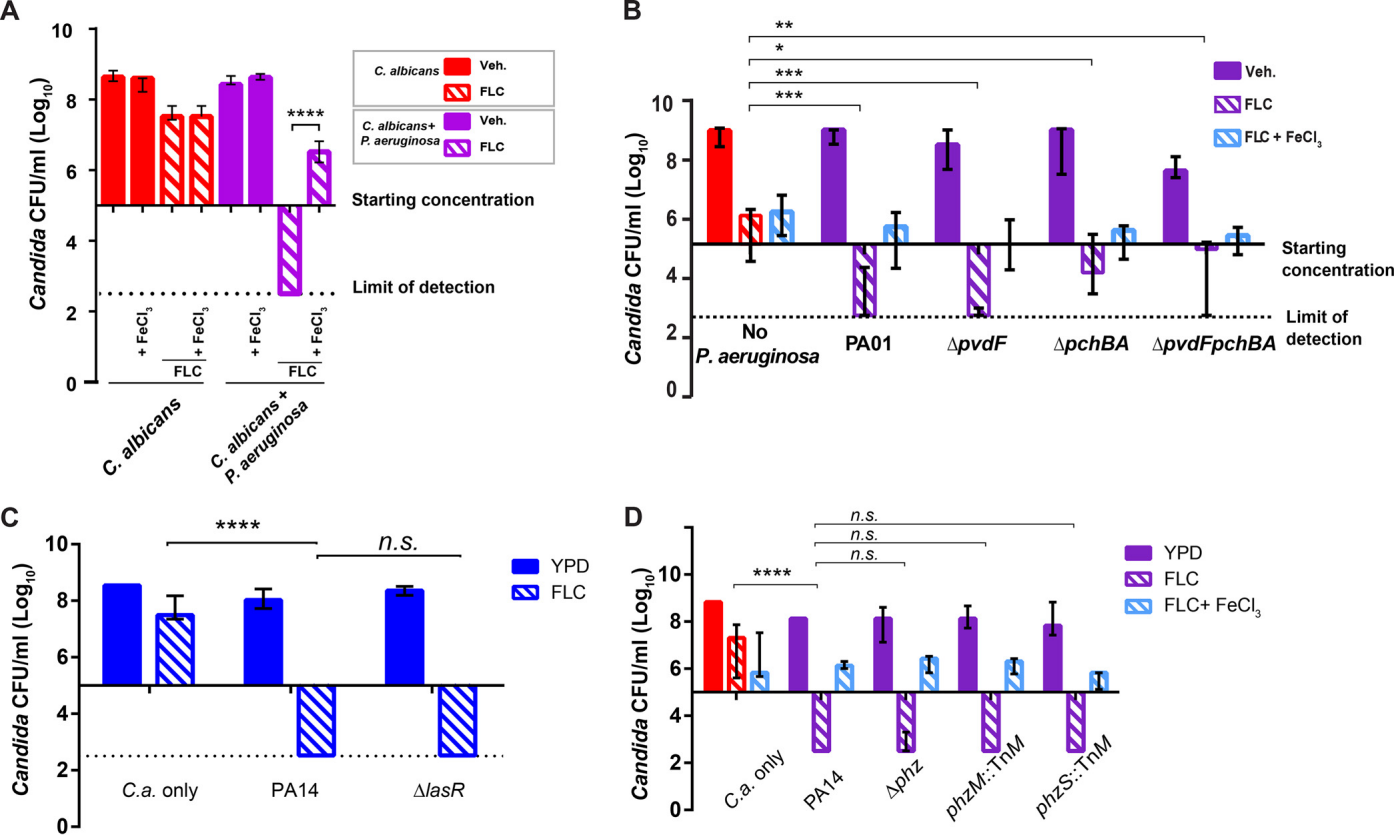
Iron supplementation partially reverses fungicidal effect *in vitro*, but phenazines and quorum sensing do not contribute to the effect. (A) FeCl_3_ supplementation reverses P. aeruginosa-FLC synergy *in vitro*. Cocultures were performed with or without FLC treatment (12.5 μg/mL) and/or FeCl_3_ (1 mM). Data from 3 independent experiments. (B) C. albicans growth after 48 h cocultures with P. aeruginosa WT or siderophore mutants: *ΔpvdF*, *ΔpchB*, *ΔpvdFpchBA*. Bar graph represents C. albicans growth in log_10_ CFU/mL. Data is representative of 4 independent experiments and medians with interquartile ranges from three independent experiments are shown. (C) Cocultures of C. albicans with P. aeruginosa WT or *ΔlasR* mutant PA14 *ΔlasR* mutant is synergistic with FLC. Bar graph represents C. albicans growth in log_10_ CFU/mL. Data is representative of 3 independent experiments. (D) Coculture of C. albicans with P. aeruginosa WT or phenazine deficient strains: PA14 Δ*phz*, PA14 *phzM::TnM*, PA14 *phzS::TnM* in the presence or absence of FLC (12.5 μg/mL). Bar graph represents C. albicans growth in log_10_ CFU/mL. Data is representative of 3 independent experiments. Data shown are the median with ranges. (*p* > 0.05 NS; < 0.05 *; <0.01 **; <0.001 ***; <0.0001 ****).

Several molecular interactions between C. albicans and P. aeruginosa play roles *in vitro* and during infection, including quorum sensing, phenazine toxins, fungal morphogenesis and iron starvation (12). Iron is an important micronutrient for both C. albicans and P. aeruginosa, and iron chelation leads to enhanced FLC activity against C. albicans (27, 38, 55). To determine if P. aeruginosa enhances FLC activity against C. albicans by outcompeting for iron, we supplemented cocultures of C. albicans and P. aeruginosa and FLC with 1 mM FeCl_3_ *in vitro*. We found that iron supplementation limits but does not eliminate the synergistic fungicidal activity of the P. aeruginosa-FLC combination (Fig. 3A). Similarly, bacteria lacking the two major siderophores pyoverdine and pyochelin had a slightly reduced ability to synergize with FLC, although the rescue was not as strong as with iron supplementation and was not affected by additional iron supplementation (Fig. 3B). The clear loss in fungal viability even upon iron supplementation is consistent with an inability for iron to restore FLC tolerance. Intriguingly, at high concentrations phenazines have been shown to have a synergistic effect with azoles against C. albicans *in vitro* (28). However, both *ΔlasR* and *Δphz* bacterial mutants had undiminished synergy with FLC against C. albicans (Fig. 3C and D). Filamentous fungal growth also does not appear to play a role, as this synergy occurs both in YPD, with >99% yeast, and in RPMI, with >50% hyphae and pseudohyphae (Fig. S2). These results indicate that P. aeruginosa synergizes with FLC *in vitro* in part by out-competing C. albicans for iron, but this synergy is largely independent of previously identified mediators of *Candida*-Pseudomonas dialog (quorum sensing, phenazine toxin production and fungal filamentous growth).

### P. aeruginosa supernatant in combination with FLC has a partial activity against C. albicans.

**FIG 4:**
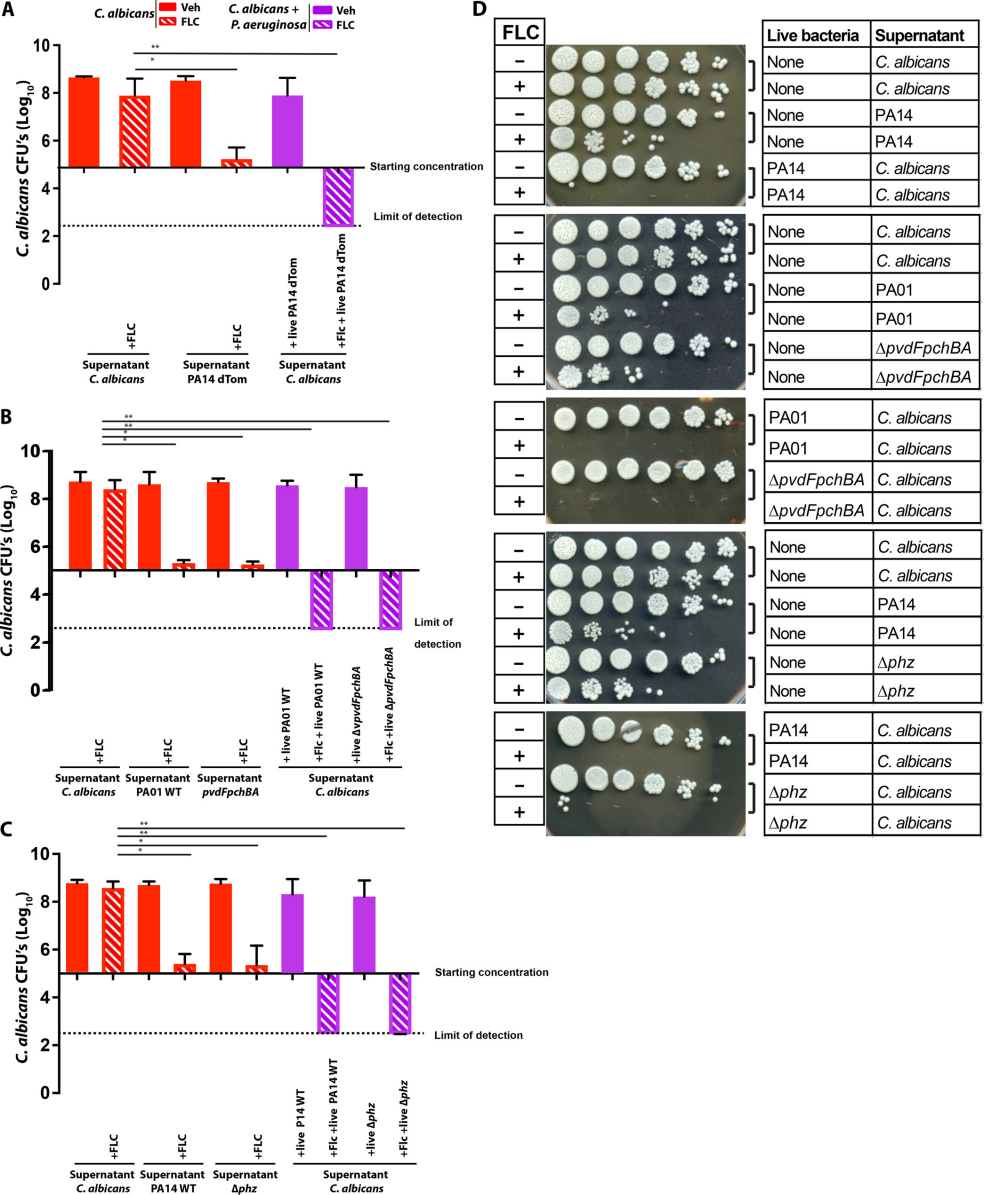
P. aeruginosa supernatants exhibit mild synergy with FLC compared to live Pseudomonas. P. aeruginosa and C. albicans were grown overnight in YPD media at 30°C. Overnight cultures supernatants were sterile filtered and added to 4 × 10^5^ C. albicans in YPD liquid media along with 12.5 μg/mL of FLC. After 48 h of incubation at 30°C, cultures were 10-fold diluted and spotted onto YPD plates with antibiotics to count CFU. (A) Supernatant from PA14-dTom strain, (B) Supernatant from PA01 WT and Δ*pvdFpchBA*, (C) Supernatant from PA14 WT and Δ*phz*, (D) Representative images of YPD plates showing the growth of C. albicans after 24 h of incubation. Data from 3 independent experiments. (*p* > 0.05 NS; < 0.05 *; <0.01 **; <0.001 ***; <0.0001 ****).

The implication of iron starvation in the interaction between C. albicans and P. aeruginosa suggested that secreted molecules might drive synergy with FLC. P. aeruginosa secretes a large number of virulence factors such as siderophores, phenazines and quorum sensing molecules that were previously shown to affect C. albicans growth (12). To test if known secreted factors contribute to the synergy seen with FLC and if they are transferable in conditioned media, we tested the activities of supernatants from WT P. aeruginosa, a double siderophore mutant and a phenazine mutant. Addition of the supernatant from P. aeruginosa did not affect C. albicans growth, whereas P. aeruginosa supernatant in combination with FLC completely blocked C. albicans trailing growth (Fig. 4A & Fig. S5). This is intermediate between FLC treatment alone, which results in trailing growth, and live P. aeruginosa, which synergizes to cause fungal death (Fig. 4A and Fig. S5). Conditioned media from C. albicans had no effect in combination with FLC, suggesting that the activity of P. aeruginosa supernatant is not due to a lack of nutrients, but rather from the activity of P. aeruginosa-secreted factors. Interestingly, the supernatant was not nearly as effective as live P. aeruginosa in synergizing with FLC. Supernatant from the double siderophore mutant and phenazine mutant strains performed indistinguishably from wildtype supernatant, inhibiting C. albicans trailing growth beyond the starting inoculum (Fig. 4B–D). Surprisingly, iron supplementation does not rescue C. albicans from the combination of FLC and P. aeruginosa conditioned media (Fig. S6). These results suggest that the full effects of P. aeruginosa-FLC synergy require live bacteria, while some activity can be transferred in conditioned media.

### Iron homeostasis plays a limited role in regulation of P. aeruginosa-mediated synergy with FLC during infection.

**FIG 5:**
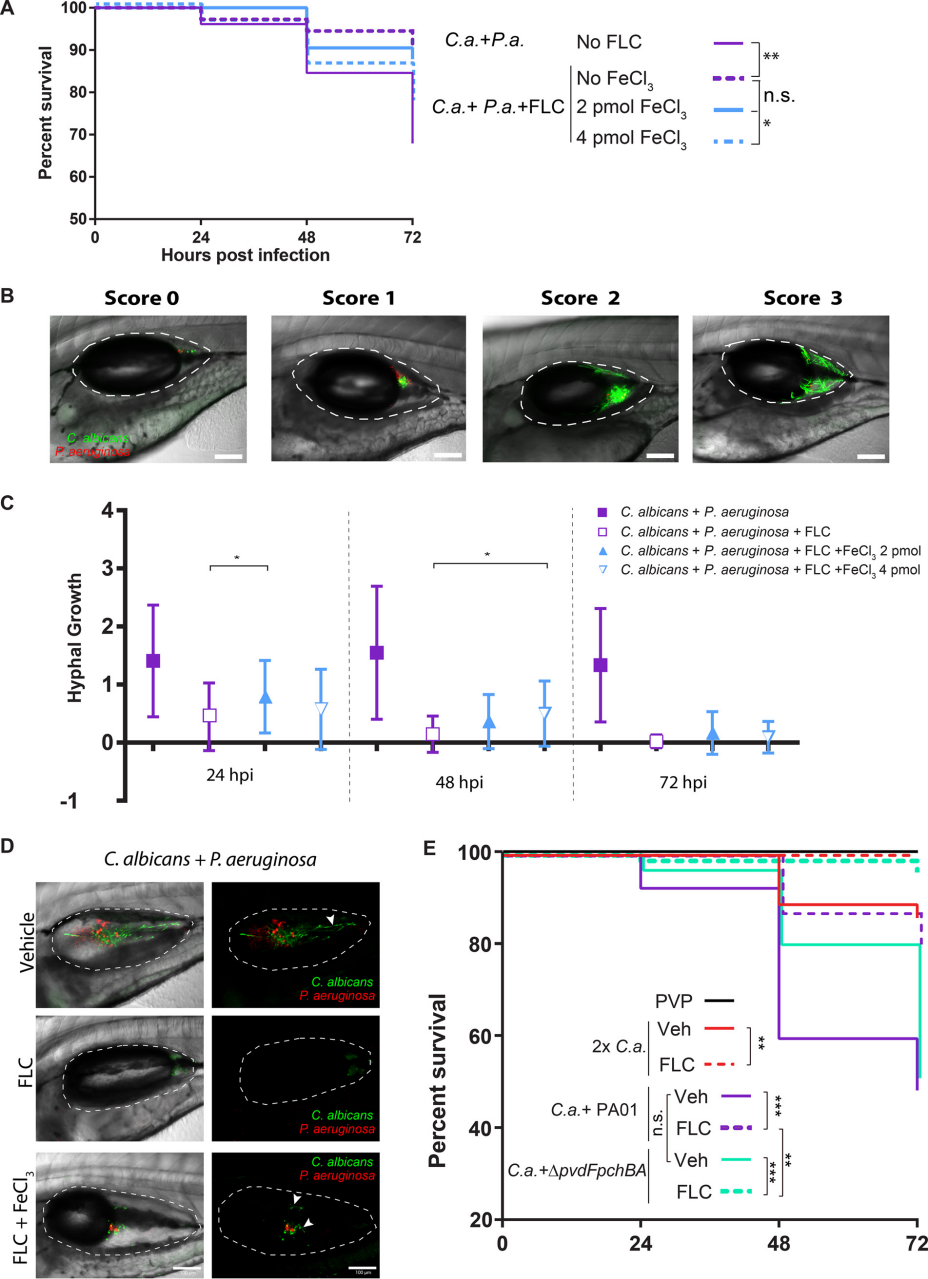
Iron homeostasis contributes to P. aeruginosa-mediated synergy with FLC during infection. (A) FeCl_3_ supplementation partially reverses P. aeruginosa-FLC synergy *in vivo*. Zebrafish injected with indicated microbes in the swimbladder with or without the indicated amounts of FeCl_3_.(2 or 4 pmol). Data pooled from 4 independent experiments. (B) Hyphal growth during infection was scored using double-blind methodology. Representative images of each score: 0-no hyphal growth; 1- < 10% coverage of swimbladder; 2- 10-50% coverage of swimbladder; 3- > 50% coverage of swimbladder. (C) FeCl_3_ supplementation is associated with stronger hyphal growth *in vivo*. Data shown are the medians with interquartile ranges from three experiments. (D) Representative images of scored hyphal growth in the swimbladder at 24 hpi. Shown are median fish from each cohort. (E) FLC treatment has no loss of effectiveness in co-infections with P. aeruginosa siderophore mutant. Scale bars = 100 μm. (*p* > 0.05 NS; < 0.05 *; <0.01 **; <0.001 ***; <0.0001 ****).

To test the contribution of iron homeostasis to FLC-P. aeruginosa synergy during infection, we again turned to the zebrafish swimbladder model. We treated co-infections with FLC, supplemented with different levels of iron, and monitored both fish survival and fungal burden. Remarkably, iron supplementation reduced the protective effects of FLC against co-infection-induced mortality in a dose-dependent manner (Fig. 5A). Imaging revealed an increase in filamentous fungi that is usually associated with virulence but would be undercounted by homogenization and plating (15, 50, 56). To quantify this type of fungal overgrowth, we used double-blind scoring of individual fish for their level of hyphal growth, classifying fish into four categories (Fig. 5B). This semi-quantitative scoring revealed a mild but significant enhancement of fungal filamentous growth upon iron supplementation (Fig. 5C). This is also seen clearly in representative images selected from fish with median scores (Fig. 5D). Consistent with both the mild effect of siderophore deletion on FLC synergy *in vitro* and the intermediate effect of iron supplementation *in vivo*, the siderophore double mutant P. aeruginosa was not hypovirulent and did not limit the effectiveness of FLC during co-infection (Fig. 5E). Taken together with our *in vitro* findings, these *in vivo* infection results argue that iron supplementation has a limited ability to reverse P. aeruginosa-FLC synergy.

## DISCUSSION

In this study, we found that fungal-bacterial interactions can drive an unexpected enhancement in antifungal susceptibility during treatment of infection. Specifically, P. aeruginosa brings out a fungicidal activity of the normally fungistatic drug fluconazole against C. albicans. We used a transparent mucosal infection model to mimic the clinical co-infections seen in cystic fibrosis and leveraged its simplicity and amenability to intravital imaging to probe the four-part interplay of two microbial species, drug therapy and host responses. These findings are clinically relevant for several reasons. First, these two microbes are frequently found together commensally and during infection, especially in cystic fibrosis. Second, there is a scarcity of effective anti-fungals, and the action of the most orally bioavailable drug is limited by tolerance, which is associated with treatment failure. Third, our results implicate iron in infection and therapy in a new way beyond strictly as a micronutrient subjected to sequestration by the host and pathogen. The ability of P. aeruginosa to modify fungal drug susceptibility *in vitro* and *in vivo* adds a new dimension to the complexities of polymicrobial infection and raises important questions about the utilization of antifungal drugs during co-infection.

The fungicidal effect of FLC during co-infection suggests that Pseudomonas blocks C. albicans tolerance to FLC, leading to death rather than persistence or slow growth during treatment. Recent work suggests that drug tolerance should be considered alongside the traditional MIC as an indicator for clinical response—and may be even more important than MIC (33). Determination of clinically-relevant drug resistance profiles in fungi is fraught with challenges and current *in vitro* testing protocols do not robustly match empirical clinical efficacy (32, 33). The disconnect between *in vitro* testing and clinical success may be due to biotic and/or abiotic factors in the host environment or may be due to a focus on the wrong metric for resistance. Microbe-microbe cross talk alters antibacterial sensitivity *in vivo* (29, 57) and may be especially relevant in chronic co-infection of the immunocompromised host (13, 31). Thus, understanding how P. aeruginosa can reduce antifungal drug tolerance during treatment of infection has potentially important implications for both diagnosis and treatment.

Manipulation of iron homeostasis by P. aeruginosa is clearly one mechanism for enhancing FLC efficacy against C. albicans. This activity may be different from the iron piracy used by P. aeruginosa against other fungi, where *in vitro* antagonism is largely transferable with soluble factors such as siderophores (24, 58, 59). We tested other potentially contributing bacterial factors, including phenazines and quorum sensing, and fungal factors, including filamentous growth, but only iron supplementation significantly modulated the live P. aeruginosa-FLC synergy. Iron starvation is known to change FLC into a fungicidal drug, perhaps by regulating membrane fluidity, limiting mitochondrial function and/or blocking calcineurin-mediated stress responses (27, 33, 38, 55, 60). However, iron supplementation only partially reverses the effect of P. aeruginosa on FLC fungicidal activity both *in vitro* and during infection, and deletion of both major siderophores has minimal effects *in vitro* and no effect *in vivo*. Thus, while it is clear that P. aeruginosa has synergy with FLC against C. albicans *in vitro* and during co-infection, iron homeostasis is only one piece of the puzzle.

Iron is an essential micronutrient for both P. aeruginosa and C. albicans that each microbe acquires by multiple pathways during infection (22, 61, 62). Iron is important for C. albicans virulence in disseminated murine candidiasis and for epithelial invasion *in vitro* (63). Further, some iron chelators can work alone or in conjunction with FLC in murine models of OPC, VVC and disseminated mucormycosis (21, 39, 64–66). Conversely, iron supplementation can enhance virulence in both our zebrafish model and in a murine GI disease model (17, 21). Nonetheless, clinical studies of iron chelation against fungal infection are inconclusive and suggest it may negatively impact health (67–69). Thus, the prospect of using iron chelation to increase drug effectiveness during treatment of human patients holds both risks and potential benefits.

C. albicans can have both positive and negative interactions with diverse bacteria, depending on the context (12, 14, 70). In co-infection, C. albicans and P. aeruginosa are synergistically virulent in both a burn model and the zebrafish mucosal model (15, 18). However, these two microbes can exhibit antagonism *in vitro* and *in vivo* (12, 71). Furthermore, interactions of C. albicans with S. epidermidis and S. aureus *in vivo* are synergistic in terms of virulence but have shown antibiotic antagonism rather than synergy (72, 73). Given these disparate results, it remains to be tested whether the P. aeruginosa-FLC synergy is broadly relevant for vertebrate co-infections with *Candida*. It will be crucial to determine which mechanisms, beyond iron homeostasis, regulate P. aeruginosa-FLC synergy *in vitro* and then test those mechanisms in zebrafish and additional infection models such as the mouse cornea (74–76).

The ability of P. aeruginosa to synergize with FLC against *Candida* is only partly transferable in conditioned media, suggesting that there are multiple bacterial contributors to this ability. Interestingly, the effect of conditioned media does not depend on iron-chelating siderophores, and iron supplementation does not reverse the effect of conditioned media. Taken together, these data suggest that siderophores and iron starvation are only effective in synergizing with FLC when live bacteria are present to scavenge the iron-replete siderophores from the media highlighting the multifactorial nature of P. aeruginosa antagonism toward *Candida*.

Co-infections of P. aeruginosa and C. albicans are infrequent, except in the context of chronically infected cystic fibrosis (CF) patients and those on a ventilator (10, 77, 78). In CF, co-isolation of both C. albicans and P. aeruginosa is associated with worse outcomes, and the risks of other co-infections and acquisition of drug resistance are higher (13, 31). Interestingly, iron levels have been shown to be increased in cystic fibrosis airways and have been implicated in facilitating P. aeruginosa infections (79). Work in a zebrafish model of CF suggests that P. aeruginosa is similarly more virulent in the absence of CFTR activity in this vertebrate, suggesting that this model may be informative in translation to human disease (80).

In summary, FLC and P. aeruginosa have a synergistic interaction against C. albicans that results in enhanced clearance of C. albicans. This increased efficacy of FLC is dependent, in part, on iron sequestration caused by P. aeruginosa. We do not yet know if other P. aeruginosa clinical isolates show similar effect or if the synergy also occurs against other *Candida* species that are intrinsically more resistant to azoles, although a similar phenomenon occurs with C. glabrata. Nonetheless, our work demonstrates that polymicrobial interactions can profoundly shift antifungal sensitivity of C. albicans. On a more general level, our results also suggest that the biotic and abiotic growth environment can influence the efficacy of antifungal drugs, pointing the way toward new strategies for developing drugs to eradicate recalcitrant infections.

## MATERIALS AND METHODS

### Ethics statement and zebrafish care and maintenance.

Adult zebrafish used for breeding embryos were housed in recirculating systems (Aquatic Habitats, Apopka, FL) at the University of Maine Zebrafish Facility. All zebrafish care protocols and experiments were performed in accordance with National Research Council guidelines (81) under Institutional Animal Care and Use Committee (IACUC) protocols A2015-11-03 and A2018-10-01. Larvae were reared at a density of 150/dish in 150-mm petri dishes containing 150 mL of E3 (5 mM sodium chloride, 0.174 mM potassium chloride, 0.33 mM calcium chloride, 0.332 mM magnesium sulfate, 2 mM HEPES in Nanopure water, pH 7) supplemented with 0.02 mg/mL of 1-phenyl-2-thiourea (PTU) (Sigma-Aldrich, St. Louis, MO) to prevent pigmentation, as well as 0.3 mg/liter methylene blue (VWR, Radnor, PA) for the first 24 h to prevent microbial growth. Larvae were manually dechorionated at 24 h postfertilization, transferred into media containing E3 and PTU, and incubated at 33°C over the course of experiments. This temperature was chosen as the highest safe temperature for zebrafish health to approximate human body temperature and is regularly used for experiments with temperature-sensitive alleles. Experiments were conducted using wild-type (AB) zebrafish.

### Strains and growth conditions.

The strains used in this study are listed in Table 1. Most experiments were conducted with the C. albicans reference strain SC5314 and either PA14 or PA01 P. aeruginosa strains. All experiments with mutant bacteria or fungi were conducted with matched controls from the source laboratory (see Table S1 for panel-by-panel description). C. albicans and P. aeruginosa strains were routinely refreshed from frozen stocks at −80°C and maintained on YPD (1% Bacto yeast extract, 2% Bacto peptone, 2% dextrose, 2% Bacto agar) plates or Pseudomonas isolation agar (Sigma-Aldrich) for *in vitro* experiments and LB agar (10 g/liter Bacto tryptone, 5 g/liter Bacto yeast extract, 10 g/liter sodium chloride, 1.2% agar; BD, San Jose, CA) supplemented with 750 μg/mL ampicillin (EMD Millipore, Billerica, MA) for injection. Liquid cultures were grown overnight in YPD or LB media in a rotator wheel at 30°C. Prior to experiments, cultures were washed with phosphate-buffered saline (PBS) and optical density OD 600 was measured.

**TABLE 1:** Fungal and bacterial strains used

Strain name	Description and Genotype	Reference
Candida albicans *and* Candida glabrata		
SC5314-Neon *C. albicans*	Wildtype clinical isolate, pENO1-NEON-NAT	(82)
Caf2-FR *C. albicans*	SC5314 background; Δ*ura3::imm434/URA3* pENO1-iRFP-NAT	(15)
SN250 *C. albicans*	*his1*Δ*/his1*Δ, *leu2*Δ*::C.dubliniensis HIS1/leu2*Δ*::C.maltosa LEU2, arg4*Δ */arg4*Δ, *URA3/ura3*Δ*::imm434, IRO1/iro1*Δ*::imm434*	(83)
*sef1* Δ/Δ *C. albicans*	*his1Δ/his1Δ, sef1Δ::C.dubliniensis HIS1/sef1Δ::C.maltosa LEU2, arg4Δ/arg4Δ, URA3/ura3Δ::imm434, IRO1/iro1Δ::imm434*	(83)
*sfu1* Δ/Δ *C. albicans*	*his1Δ/his1Δ, sfu1Δ::C.dubliniensis HIS1/sfu1Δ::C.maltosa LEU2, arg4Δ/arg4Δ, URA3/ura3Δ::imm434, IRO1/iro1Δ::imm434*	(83)
NCO-788 *C. albicans*	Clinical isolate	(84) (Clancy and Shields, U. Pittsburgh)
NC1 C. glabrata	Clinical isolate	(84) (Clancy and Shields, U. Pittsburgh)
NC999 C. glabrata	Clinical isolate	(84) (Clancy and Shields, U. Pittsburgh)
CG-4720 C. glabrata	Clinical isolate	(85)
B13-TWO7229#2 *C. albicans*	Clinical isolate #2 in series from patient.	(84, 86)
B14-TWO7230#3 *C. albicans*	Clinical isolate #3 in series from patient	(84, 86)
B15-TWO7241#16 *C. albicans*	Clinical isolate #16 in series from patient	(84, 86)
B16-TWO7243#17 *C. albicans*	Clinical isolate #17 in series from patient	(84, 86)
Pseudomonas aeruginosa		
PA14 dTom	PA14 carrying plasmid encoding dTomato	(80)
PA14 Δ*lasR*	In‐frame deletion of *lasR*	(87)
PA01 WT	Wild type clinical isolate	(88)
PAO6382	PA01 Δ*pvdF*	(89)
PAO6297	PA01 Δ*pchBA*	(90)
PAO6383	PA01 Δ*pvdFΔpchBA*	(90)
PA14 WT	Wildtype clinical isolate	(91)
PA14 Δ*phz*	In-frame deletion of *phzA1-G1* and *phzA2-G2* operons, phenazine negative	(91)
PA14 *phzM::TnM*	TnM mutant, 5-MPCA negative	(91)
PA14 *phzS::TnM*	TnM mutant, PYO negative	(91)

### *In vitro* C. albicans and P. aeruginosa coculture.

P. aeruginosa and C. albicans were individually grown overnight. P. aeruginosa was grown in GGP media (3% glycerol, 1% proteose peptone, 2.9 mM K_2_HPO_4_, 2 mM MgSO_4_●7H_2_0) or LB media (10 g/liter Bacto tryptone, 5 g/liter Bacto yeast extract, 10 g/liter sodium chloride; BD, San Jose, CA) at 30°C. C. albicans was grown in YPD media at 30°C. P. aeruginosa and C. albicans cultures were combined in a 1:1 ratio with both organisms at a final concentration of 2 × 10^5^/mL. The P. aeruginosa*/*C. albicans coculture was grown at 30°C for 48 h in YPD on a rotating wheel. Fluconazole (Sigma-Aldrich) was used at 12.5 μg/mL unless specified. This is significantly above the MIC_50_ for strains and limits any experimental variability due to slight differences in drug concentration. The spot test was performed by spotting 3 μL from each dilution using a multichannel pipette, plated on YPD agar supplemented with penicillin (250 U/mL)-streptomycin (250 μg/mL) (Lonza), 30 μg/mL gentamicin sulfate (BioWhittaker, Lonza), and 3 μg/mL vancomycin hydrochloride (Amresco, Solon, OH) and on Pseudomonas isolation agar (Sigma-Aldrich) for C. albicans and P. aeruginosa selection respectively. Plates were incubated for 24 h at 37°C.

For collecting supernatants, P. aeruginosa overnight cultures in LB or GGP were centrifuged at 21,000 × *g* for 2 min and supernatant was filtered using an Acrodisc 0.2 μm syringe filter (PALL corporation). Filtered supernatant was added to 4 × 10^5^ C. albicans in YPD liquid media. 48 h post incubation, cultures were 10-fold diluted and spot tests were performed as described above.

### Swimbladder infections via microinjection.

At 4 days postfertilization, zebrafish larvae were anesthetized in Tris-buffered tricaine methane sulfonate (160 μg/mL; Tricaine; Western Chemicals, Inc., Ferndale, WA) and selected for swimbladder inflation. Fish were microinjected as previously described (48). Fungal and bacterial cells were resuspended in 5% polyvinylpyrrolione (PVP; Sigma-Aldrich) and fish were injected with 4 nl of PVP control, C. albicans at 5 × 10^7^ CFU/mL, or a C. albicans*-*P. aeruginosa mixture at 2.5 × 10^7^ CFU/mL for each. The C. albicans*-*P. aeruginosa coculture was prepared by combining equal volumes of C. albicans at 5 × 10^7^ CFU/mL and P. aeruginosa at 5 × 10^7^ CFU/mL prior to injection. As indicated, FeCl_3_ (Sigma-Aldrich) was added to the injection solution with C. albicans and/or P. aeruginosa to a final concentration of 0.5 mM or 1 mM, for a final amount of 2 or 4 pmol per 4 nl dose. Within 1 h of injection, larvae were placed in individual wells of a 96-well glass-bottom imaging dish (Greiner Bio-One, Monroe, NC) and screened for an inoculum of 50 to 100 yeast cells for mono-infection, and 25 to 50 yeast cells for co-infection, using a Zeiss AxioVision VivaTome microscope. For mortality experiments, fish were kept at 33°C in E3 containing PTU with or without fluconazole at 100 μg/mL. Fish were held for 3 days post injection and monitored daily for survival.

### Confocal laser scanning fluorescence microscopy.

At 24, 48, and 72 h post-injection, larvae were anesthetized in Tricaine and immobilized in 0.4% low-melting-point agarose (Lonza, Switzerland) in E3 containing Tricaine in a 96-well glass-bottom imaging dish (Greiner Bio-One, Monroe, NC). Confocal images were acquired using an Olympus IX-81 inverted microscope with an FV-1000 laser scanning confocal system (Olympus, Waltham, MA). The EGFP, dTomato, and Far-Red fluorescent proteins were detected by laser/optical filters with a 20× (NA, 0.7) for excitation/emission at 488 nm/505 to 525 nm, 543 nm/560 to 620 nm, and 635 nm/655 to 755 nm, respectively. Z-stacks of 15 to 25 slices, with an interslice interval between 7 and 13 μm, were collected and processed using FluoView (Olympus, Waltham, MA).

### Image analysis.

The percentage of the swimbladder covered by *Candida* at 24, 48, and 72 hpi was determined using Fiji software (ImageJ) applied to maximum-projection images from stacks of 15 to 25 z-slices. Images were taken with identical acquisition settings to ensure comparability. The swimbladder area was delineated, and the percent coverage of *Candida* fluorescence above a set threshold (corresponding to background fluorescence) was calculated. Images covered the swimbladder from midline to skin in 5-μm z-slices. The number of slices per image ranged from 15 to 25, depending on the size of the fish.

### Hyphal growth scoring from confocal images.

Zebrafish infected with C. albicans SC5314-Neon and PA14-dTomato with or without Fluconazole. FeCl_3_ (2 or 4 pmol) was co-injected into swimbladder along with C. albicans *and* P. aeruginosa. Fish were imaged at 24, 48, and 72 hpi and images were processed as described above. Hyphal growth of C. albicans in the swimbladder was scored blindly as follows: 0 = no hyphal growth; 1 = <10% hyphal growth; 2 between 10 and 50% hyphal growth; and 3  = >50% hyphal growth.

### CFU assessments.

For CFU quantification, 5 randomly selected infected larvae were pooled and homogenized at 24, 48, and 72 hpi in 500 μL of 1X PBS. For plating, 50 μL or 100 μL of homogenate from groups was plated on both YPD agar supplemented with 250 U/mL, 250 μg/mL penicillin-streptomycin (Lonza), 30 μg/mL gentamicin sulfate (BioWhittaker, Lonza), and 3 μg/mL vancomycin hydrochloride (Amresco, Solon, OH) and on Pseudomonas isolation agar (Sigma-Aldrich) for C. albicans and P. aeruginosa selection, respectively. Plates were incubated overnight at 37°C, colonies were counted the following day, and CFU/fish was calculated.

### Statistical analyses.

Statistical analyses were conducted using GraphPad Prism 7 software (GraphPad Software, Inc., La Jolla, CA). Data was analyzed for normality and appropriate parametric or nonparametric tests were performed, means or medians are shown, respectively. All significant differences are indicated in the figures, with *, **, ***, and **** indicating *P* values of <0.05, <0.01, <0.001, and <0.0001, respectively. Kaplan-Meier survival curves were subjected to a log rank (Mantel-Cox) test, and Bonferroni correction was then used to determine statistical differences between pairs of treatments. Monte-Carlo simulation was used to analyze ratios in Fig. 2D. Mann-Whitney test was used to analyze experiments in figure panels 2C, 2E, 3A, 5C. Unpaired T-test was used for Fig. 2E Two-way ANOVA was used for Fig. 3B to D. For Fig. 4A–C, significance was established by identifying non-overlapping 95% confidence intervals.
