# Supplementary material for: Pseudomonas Synergizes with Fluconazole against Candida during Treatment of Polymicrobial Infection
Source: Infect Immun. 2022 Mar 15;90(4):e00626-21. doi: 10.1128/iai.00626-21 (PMC9022521; doi:10.1128/iai.00626-21)
Supplement: SUPPLEMENTAL FILE 1 — Supplemental material. Download iai.00626-21-s0001.pdf, PDF file, 9.5 MB [file iai.00626-21-s0001.pdf]

Pseudomonas synergizes with fluconazole against _Candida_ during treatment of polymicrobial infection

Siham Hattab[1], Anna-Maria Dagher[1], Robert T. Wheeler[1,2,*]

[1]Department of Molecular & Biomedical Sciences, University of Maine, Orono, ME 04469
[2]Graduate School of Biomedical Sciences and Engineering, University of Maine, Orono, ME 04469

[*]Correspondence:
Robert T. Wheeler
5735 Hitchner Hall
Orono, ME 04469
robert.wheeler1@maine.edu
207-581-2890

Running title: *Pseudomonas*-fluconazole anti-*Candida* synergy

Supplemental Information (Methods, Tables, Figures)

Supplemental Methods

Statistical analyses. Statistical analyses were conducted using GraphPad Prism 7 software (GraphPad Software, Inc., La Jolla, CA). Data was analyzed for normality and appropriate parametric or nonparametric tests were done; means or medians are shown, respectively. All significant differences are indicated in the figures, with *, **, ***, and **** indicating P values of <0.05, <0.01, <0.001, and <0.0001, respectively. Kaplan-Meier survival curves were subjected to a log rank (Mantel-Cox) test, and Bonferroni correction was then used to determine statistical differences between pairs of treatments. One-way ANOVA was used to analyze Fig. S1, S2 and S4. For Fig. S5 and Fig. S6, significance was established by identifying non-overlapping 95% confidence intervals.

Supplemental Tables and Figures

Supplemental Table 1. Relevant measured MIC_{50} for clinical strains

Clinical isolate strain	FLC MIC_{50} as measured here
NCO-788	200 µg/ml
B13 TWO7229#2	50 µg/ml
B14 TWO7230#3	50 µg/ml
B15 TWO7241#16	100 µg/ml
B16 TWO7243#17	400 µg/ml
SN250 (WT)	12.5 µg/ml
NC1 *C. glabrata*	50 µg/ml
NC999 *C. glabrata*	400 µg/ml
CG-4720 *C. glabrata*	200 µg/ml

Supplemental Figure Legends

Fig. S1. Heat killed *Pseudomonas aeruginosa* does not synergize with FLC *in vitro*.
P. aeruginosa was heat killed by incubating at 100° C for 20 min. Heat killed *P. aeruginosa* was adjusted to 2 x 10^7/ml in YPD and added to 2 x 10^5/ml of *C. albicans*. Co-cultures were incubated at 30° C for 48 hr. 3 µl of serial 10-fold dilutions of co-cultures were plated on YPD agar plate containing antibiotics or on PIA plates selective for *P. aeruginosa*. Data shown are the medians with ranges for three experiments.

Fig. S2. *P. aeruginosa* also synergizes with FLC at 37°C and in YPD + FBS. (A) Fluconazole (FLC) treatment of *C. albicans* and *P. aeruginosa* co-culture shows a fungicidal effect in YPD at 37° C. *P. aeruginosa* and *C. albicans* were inoculated at 2x10^5/ml in YPD, FLC was added at 12.5 µg/ml. After incubation of liquid co-cultures at 37℃ for 48 hr, 3 µl of serial 10-fold

dilutions of co-cultures were plated on YPD containing antibiotics. Plates were grown at 37° C for 24 hr. Data shown are the medians with ranges for three experiments. (B-C) Fluconazole (FLC) treatment of *C. albicans* and *P. aeruginosa* shows a fungicidal effect after co-culture in YPD + 2% FBS at 37° C. *P. aeruginosa* and *C. albicans* were inoculated at $2x10^5$/ml in YPD + 2% FBS. FLC was added at 12.5 µg/ml and liquid cultures incubated at 37°C for 48 hr. (B) 3 µl of serial 10-fold dilutions of co-cultures were plated on YPD containing antibiotics. Plates were grown at 37° C for 24 hr before counting. Data shown are the medians with ranges from three experiments. (C) Representative images_showing *C. albicans* morphology as yeast in YPD or hyphal growth in YPD + 2% FBS. After 48 hr of co-culture, 5 µl of co-culture was imaged using Zeiss Vivatome microscope. White arrowheads show hyphal growth in YPD +2 % FBS. Black arrowheads show swollen cells associated with trailing growth in FLC. Arrows note dead *C. albicans* cells in both YPD and YPD + 2% FBS conditions in co-cultures with FLC.

Fig. S3. FLC does not affect larval zebrafish survival. Zebrafish larvae (4 dpf) were anesthetized in Tris-buffered tricaine methane sulfonate and selected for swimbladder inflation. Fish were microinjected with a 4 nl of PVP control. Fish were kept at 33°C in E3 containing PTU with or without FLC at 100 µg/ml. Fish were held for 3 days post injection and monitored daily for survival.

Fig. S4. FLC does not affect *P. aeruginosa* growth *in vitro*. Addition of FLC to *P. aeruginosa* alone and in co-culture with *C. albicans* does not affect *P. aeruginosa* growth *in vitro*. *P. aeruginosa* and *C. albicans* were inoculated at $2x10^5$/ml in YPD, FLC was added at 12.5 µg/ml. Cultures were grown at 30° C for 48 hr. 3 µl of serial 10-fold dilutions of co-cultures were plated

on *Pseudomonas* isolation agar (PIA). Graph showing log_{10} CFU/ml for *P. aeruginosa* viability. Data shown are the median with ranges from 3 experiments.

Fig. S5. *P. aeruginosa* supernatant affect *C. albicans* trailing growth in the presence of FLC

Addition of *P. aeruginosa* supernatant is not as effective as live *P. aeruginosa* in inhibiting *C. albicans* growth, but supernatant from both PA14 WT and PA14 Δ*phz* causes inhibition of trailing growth past the starting inoculum. Graph showing *C. albicans* viability after 24 hr and 48 hr of growth in the presence of FLC, with *C. albicans* or *P. aeruginosa* supernatant, with or without live *P. aeruginosa*. Data show medians and 95% confidence intervals from 3 experiments, with comparable data from other experiments with siderophore mutants.

Fig. S6. $FeCl_3$ supplementation does not block fungicidal synergy of *P. aeruginosa* combined with FLC.

$FeCl_3$ supplementation reverses fungicidal activity of live *P. aeruginosa* + FLC, but not when added to *P. aeruginosa* supernatant + FLC. Supernatant from overnight cultures of either *C. albicans* or *P. aeruginosa* grown in YPD was filter-sterilized, and added at 1:1 dilution to YPD with *C. albicans* at $2x10^5$/ml. FLC was added at 12.5 μg/ml and different concentrations of $FeCl_3$ used range from 1 mM to 0.125 mM. Data are representative of 3 independent experiments. Graphs show medians with 95% confidence intervals.

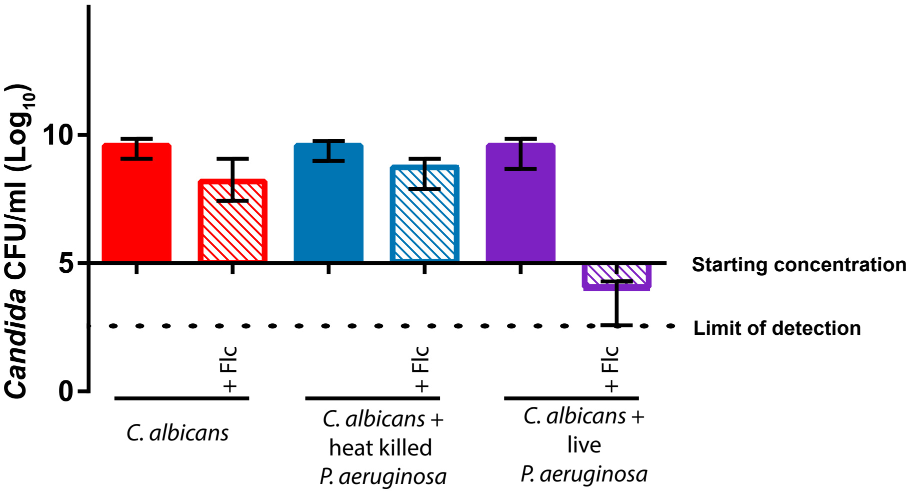
Candida CFU/ml (Log10)
10
5
0
Starting concentration
Limit of detection
+ Flc
+ Flc
+ Flc
C. albicans
C. albicans +
heat killed
P. aeruginosa
C. albicans +
live
P. aeruginosa

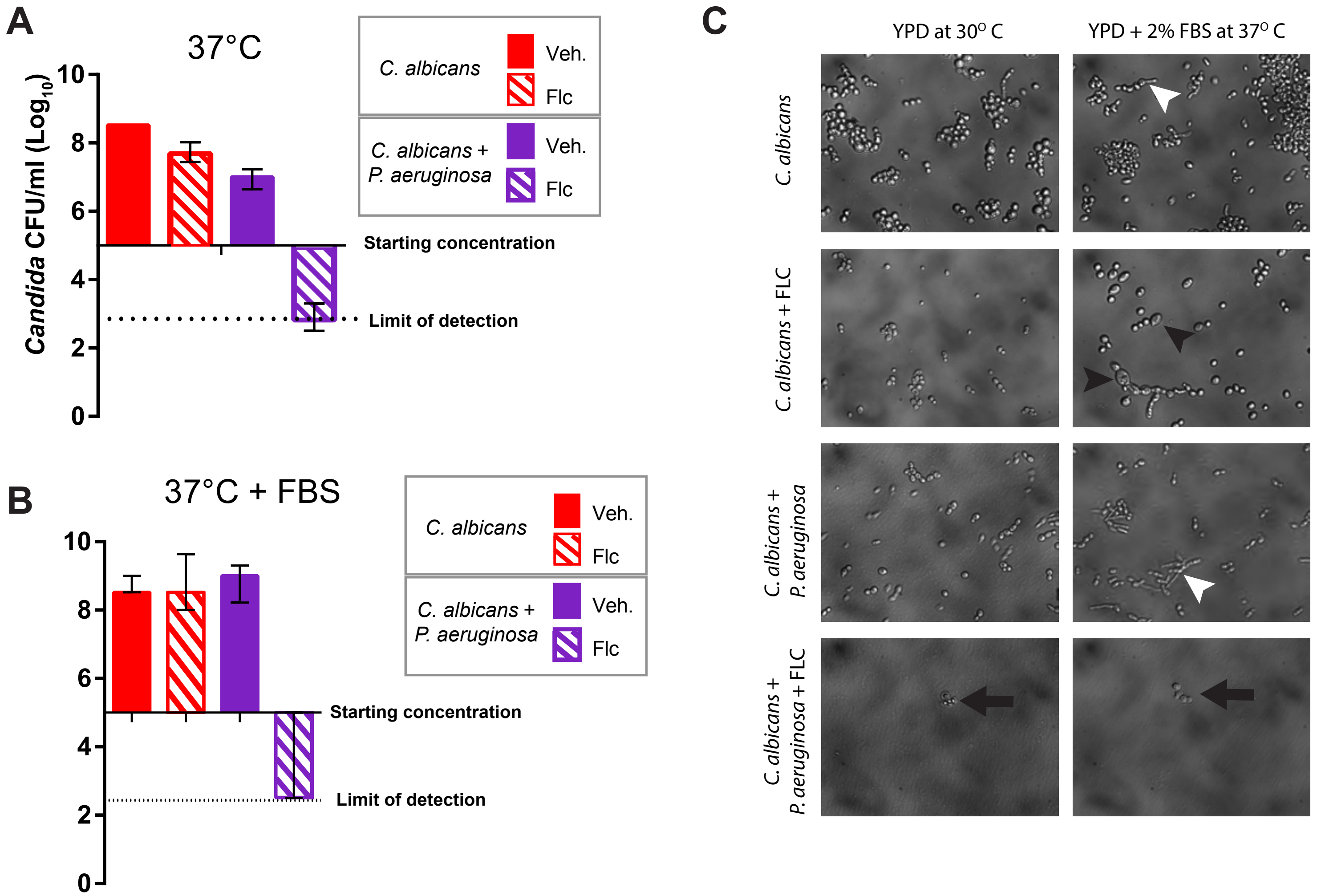
A
37°C
Candida CFU/ml (Log10)
10
8
6
4
2
0
C. albicans
Veh.
Flc
C. albicans +
P. aeruginosa
Veh.
Flc
Starting concentration
Limit of detection
B
37°C + FBS
10
8
6
4
2
0
C. albicans
Veh.
Flc
C. albicans +
P. aeruginosa
Veh.
Flc
Starting concentration
Limit of detection
C
YPD at 30° C
YPD + 2% FBS at 37° C
C. albicans
C. albicans + FLC
C. albicans +
P. aeruginosa
C. albicans +
P. aeruginosa + FLC

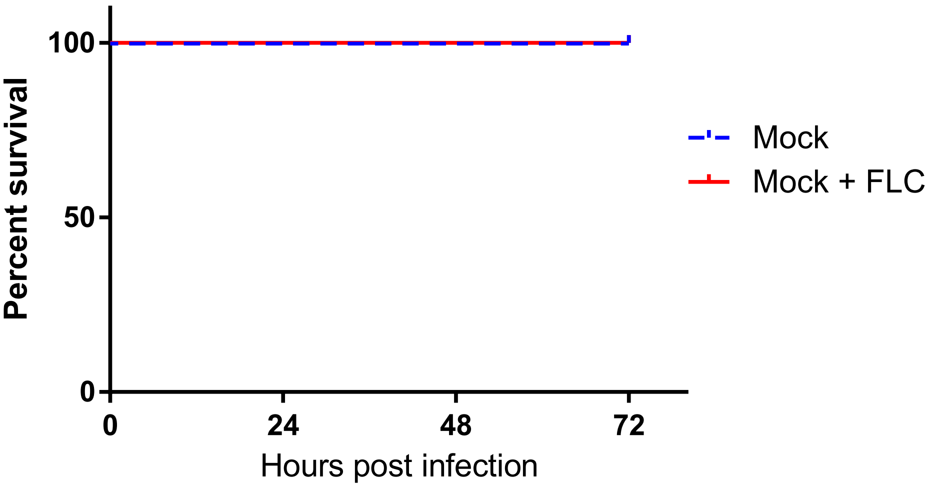

Percent survival
100
50
0
0
24
48
72
Hours post infection
Mock
Mock + FLC

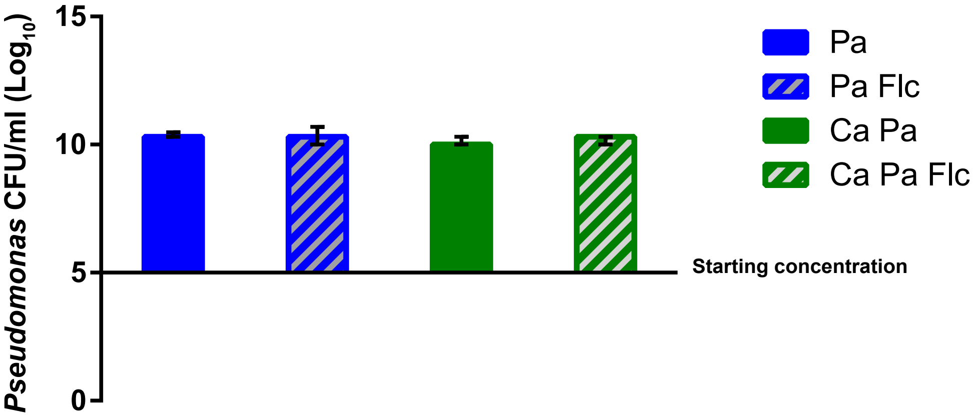

Pseudomonas CFU/ml (Log10)
15
10
5
0
Starting concentration
Pa
Pa Flc
Ca Pa
Ca Pa Flc

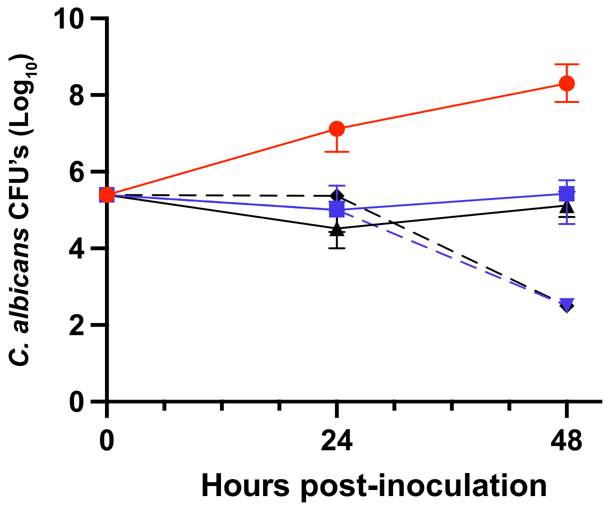

FLC	Live bacteria	Supernatant
+	None	*C. albicans*
+	None	PA14
+	None	Δ*phz*
+	PA14	*C. albicans*
+	Δ*phz*	*C. albicans*

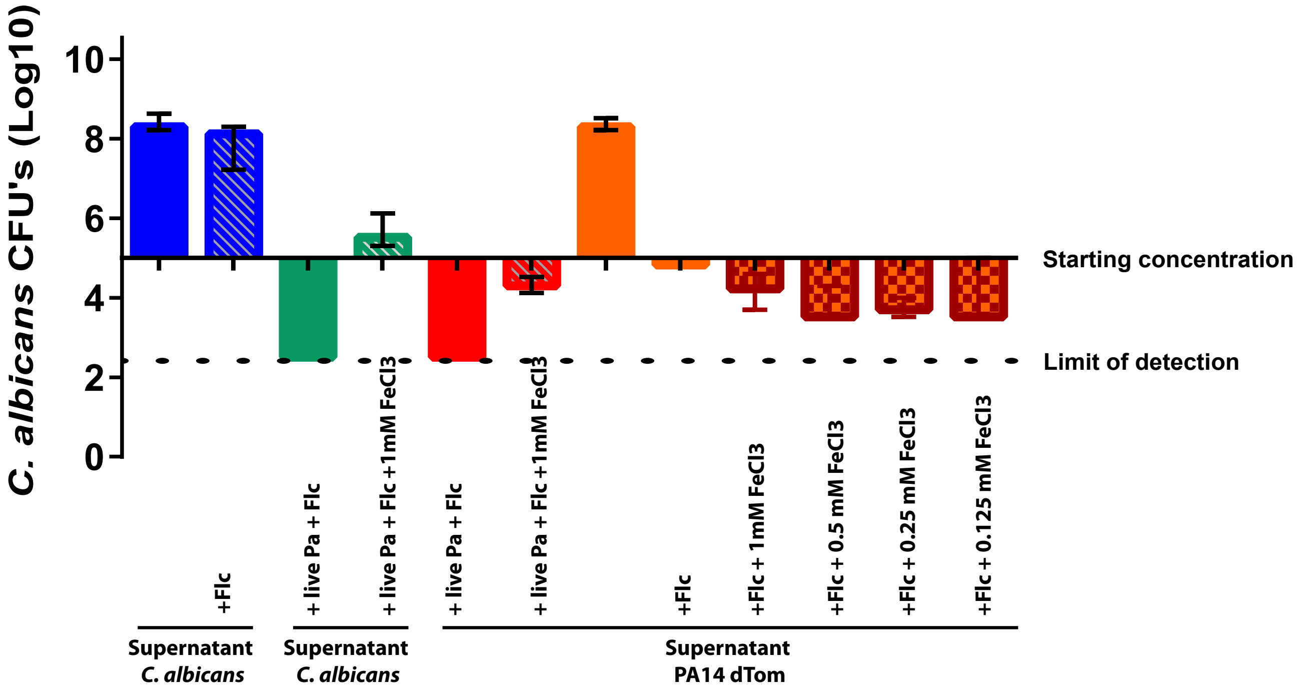
C. albicans CFU's (Log10)
10
8
6
4
2
0
Starting concentration
Limit of detection
+Flc
+ live Pa + Flc
+ live Pa + Flc +1mM FeCl3
+ live Pa + Flc
+ live Pa + Flc +1mM FeCl3
+Flc
+Flc + 1mM FeCl3
+Flc + 0.5 mM FeCl3
+Flc + 0.25 mM FeCl3
+Flc + 0.125 mM FeCl3
Supernatant C. albicans
Supernatant C. albicans
Supernatant PA14 dTom

Fig. S8
